# Intercalating Electron Dyes for TEM Visualization of DNA at the Single‐Molecule Level

**DOI:** 10.1002/cbic.201800638

**Published:** 2019-02-07

**Authors:** Yoones Kabiri, Alessandro Angelin, Ishtiaq Ahmed, Hatice Mutlu, Jens Bauer, Christof M. Niemeyer, Henny Zandbergen, Cees Dekker

**Affiliations:** ^1^ Kavli Institute of Nanoscience Delft Delft University of Technology Van der Maasweg 9 2629 HZ Delft The Netherlands; ^2^ Karlsruhe Institute of Technology (KIT) Institute for Biological Interfaces (IBG-1) Hermann-von-Helmholtz-Platz 76344 Eggenstein-Leopoldshafen Germany; ^3^ Karlsruhe Institute of Technology (KIT) Soft Matter Synthesis Laboratory Institute for Biological Interfaces (IBG-3) Hermann-von-Helmholtz-Platz 76344 Eggenstein-Leopoldshafen Germany; ^4^ Karlsruhe Institute of Technology (KIT) Macromolecular Architectures Institut für Technische Chemie und Polymerchemie Engesserstrasse 18 76128 Karlsruhe Germany

**Keywords:** DNA, contrast agents, intercalation, single-molecule studies, scanning transmission electron microscopy

## Abstract

Staining compounds containing heavy elements (electron dyes) can facilitate the visualization of DNA and related biomolecules by using TEM. However, research into the synthesis and utilization of alternative electron dyes has been limited. Here, we report the synthesis of a novel DNA intercalator molecule, bis‐acridine uranyl (BAU). NMR spectroscopy and MS confirmed the validity of the synthetic strategy and gel electrophoresis verified the binding of BAU to DNA. For TEM imaging of DNA, two‐dimensional DNA origami nanostructures were used as a robust microscopy test object. By using scanning transmission electron microscopy (STEM) imaging, which is favored over conventional wide‐field TEM for improved contrast, and therefore, quantitative image analysis, it is found that the synthesized BAU intercalator can render DNA visible, even at the single‐molecule scale. For comparison, other staining compounds with a purported affinity towards DNA, such as dichloroplatinum, cisplatin, osmium tetroxide, and uranyl acetate, have been evaluated. The STEM contrast is discussed in terms of the DNA–dye association constants, number of dye molecules bound per base pair, and the electron‐scattering capacity of the metal‐containing ligands. These findings pave the way for the future development of electron dyes with specific DNA‐binding motifs for high‐resolution TEM imaging.

## Introduction

Unstained DNA and related biomolecules contain mostly light elements, such as carbon, nitrogen, and phosphorous, that all have low electron‐scattering strengths. As a result, high‐resolution TEM imaging of unstained single DNA molecules supported on commercial carbon supports has been unsuccessful because, as the electron wave passes through DNA, only negligible changes occur to its amplitude or phase, which consequently leaves the unstained DNA invisible under TEM. The use of electron dyes is therefore a stringent requirement for increasing electron scattering, and hence, the TEM visualization of DNA.

Surprisingly, despite the pressing need for DNA‐staining compounds, there is a historical gap in the systematic investigation of effective and accessible electron dyes. Uranyl acetate (UA) and its analogues have been the only DNA stain since 1961.^1^ UA binds covalently to the negatively charged backbone of DNA,^1^ and with the heavy atomic number of 92 for the uranium element, it provides excellent electron scattering in TEM. Practically, the use of UA is not ideal because it is extremely toxic and its access requires governmental permissions due to tight restrictions on nuclear fuel materials. It is well known that the DNA‐binding mode, and consequently, cellular response to compounds changes with even slight modifications in the coordination chemistry of the transition metals.^2^ Hence, from a biochemistry point of view, it remains an interesting question whether it would be possible to engineer a synthetic compound with a compatible uranium core element for providing excellent TEM contrast, but with an intercalation binding mode, rather than covalent attachment to the DNA phosphate backbone.

Can intercalation be utilized for TEM imaging of DNA? The design or application of intercalating molecules containing heavy elements as electron dyes has been very limited so far. For example, although many DNA‐binding compounds that contain heavy elements (Pt, Ag, Au, etc.) are conceivable, only platinum has gained attention due to its rich library of coordination chemistry. Indeed, since the first successful application of cisplatin as an anticancer agent, the field of synthetic platinum compounds has evolved exponentially.^2^, ^3^ Cisplatin is a covalent DNA binder, whereas other platinum complexes, especially those containing planar aromatic terpyridine moieties, are intensively studied DNA intercalators, owing to their vast therapeutic applications in chemotherapy and cancer treatment.^3^, ^4^, ^5^, ^6^ In addition, it has recently been shown that some derivatives of platinum complexes can penetrate into the cell nucleus and show cell viability.^4^, ^6^, ^7^ This grants them a clear advantage for TEM imaging applications of biological samples. However, despite these promising intercalation properties, no TEM visualization of single DNA molecules has been reported, so far, through intercalation binding of platinum or any other heavy elements, such as uranium.

Two major drawbacks have impeded progress towards the systematic investigation of contrast agents for TEM visualization of DNA: 1) demanding and sometimes ambiguous sample preparations, and 2) nonoptimized choices for the TEM imaging mode. With regard to the first point, most reports in the literature use plastic‐embedded tissue sections or viruses as the model system for microscopic investigations.^8^, ^9^ Apart from tedious sample preparation, artifacts often affect the results due to the crowded environment (presence of proteins, lipids, DNAs, etc.)^10^ The second drawback is associated with inherently noisy TEM images, even for stained DNA. Due to the faint signal of DNA, contaminants or the substrate signal often interfere with imaging and reduce the obtained DNA contrast. Accordingly, reliable and quantitative analysis of DNA images has proven to be difficult and sometimes impossible.

**Scheme 1:**
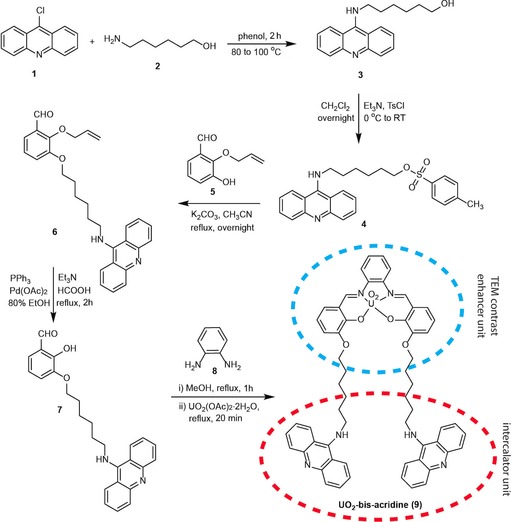
Synthetic scheme for the preparation of BAU. The complete protocols for the synthesis are given in the Supporting Information.

**Figure 1:**
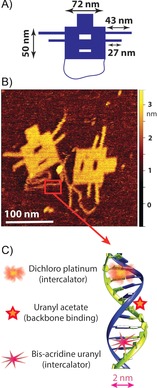
DNA origami structures as robust and straightforward microscopic model specimens to probe various TEM contrast agents for DNA visualization. A) Schematic representation of our 2D DNA origami design with various features, such as cavities in the main rectangle, side arms (2 DNA helices, 4 nm wide), and an individual double‐stranded (ds) DNA loop at the bottom (2 nm wide). B) Liquid‐cell AFM images of the DNA nanoplates on mica. C) Schematic illustration of the binding sites for the compounds discussed herein.

Here, we present a novel intercalating agent that is synthesized by covalently tethering a bis‐acridine moiety to a salophen ligand, that is, a Schiff base, which functions as a chelator for a uranium atom (Scheme 1). We fully characterized our synthesized intercalator by means of NMR spectroscopy, MS, and gel electrophoresis. We then present our TEM investigations. To overcome sample preparation problems, we utilized a well‐defined DNA origami nanostructure as a microscopic test model system for TEM imaging (Figure 1). DNA origami, that is, DNA folded into a user‐defined shape,^11^ provides a facile and straightforward approach to evaluate stained DNA and strongly reduces the above‐mentioned sample preparation challenges because the characteristic shape of the origami design facilitates the detection and quantitative analysis of the DNA contrast. Furthermore, we significantly improve TEM imaging by using the scanning transmission electron microscopy (STEM) technique. STEM is advantageous over conventional wide‐field TEM because it provides a higher signal‐to‐noise ratio (SNR) for single‐image acquisitions. Indeed, STEM enabled us to quantitatively analyze the DNA contrast stained with different DNA‐binding electron dyes, including our synthesized bis‐acridine uranyl (BAU) and other compounds reported in the literature, such as the dichloroplatinum (DP; Pt intercalator), cisplatin, and osmium tetroxide.

## Results

### Synthesis, characterization, and binding properties of BAU to DNA

Intercalators are widely applied in analytical and medical chemistry,^12^ and they offer the potential for tailoring the surface properties of DNA nanostructures.^13^ Inspired by the wide range of useful properties of acridine derivatives, and in continuation of efforts to expand its applications as staining agents, we synthesized a novel bidentate intercalator compound, BAU (Scheme 1), that contains one uranyl cation confined inside the chelating salophen moiety in the center of its structure, which acts as a TEM contrast enhancer. This unit is tethered to two acridine heterocycles (bis‐acridine) that serve as DNA intercalating ligands. The linker strategy for tethering the bis‐acridine moiety to the uranium metal center was adopted from salophen‐type coordination chemistry.^14^ A full description of the experimental procedures for the synthesis of BAU is given in the Experimental Section and the Supporting Information.

**Figure 2:**
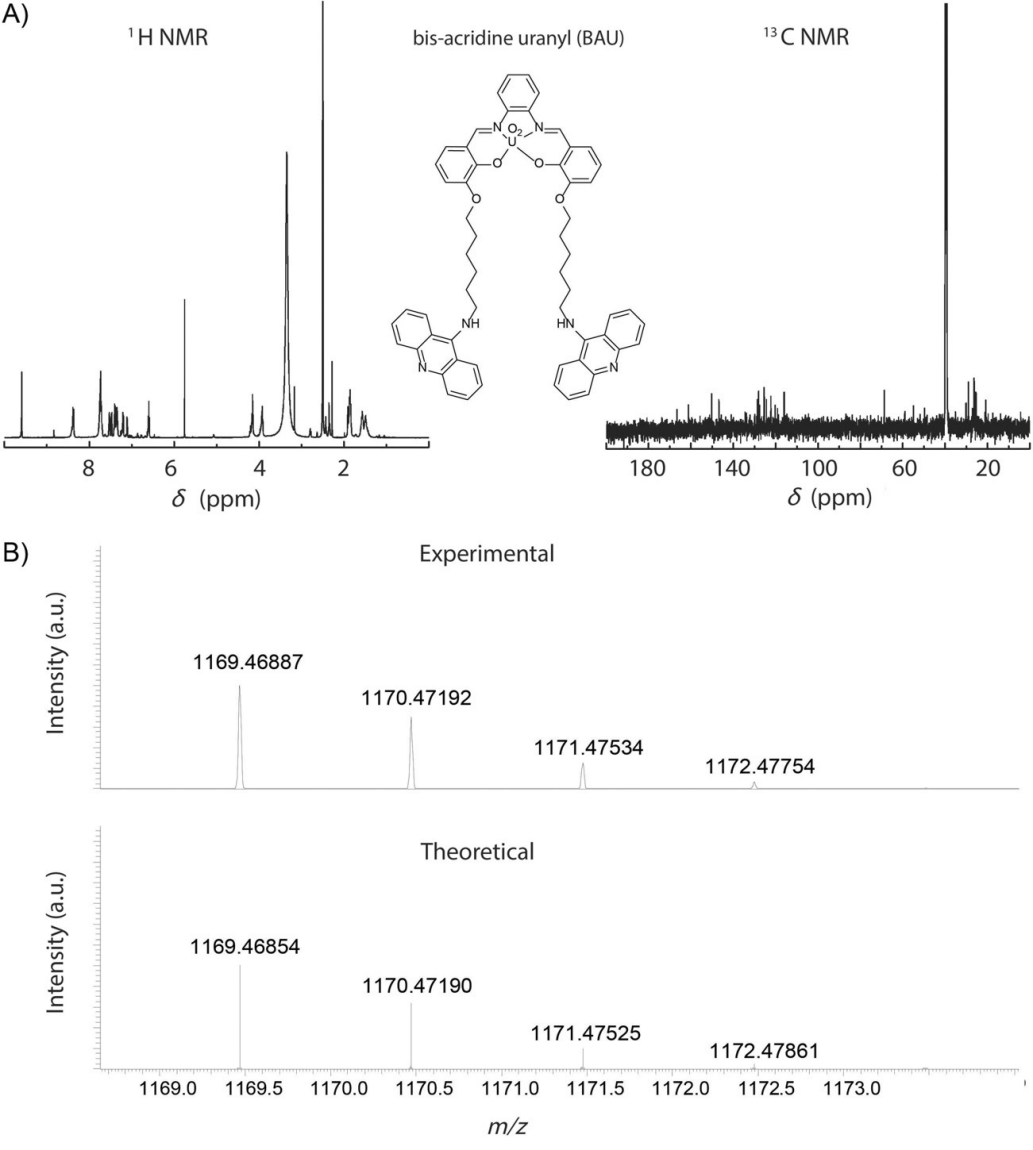
A) 1D NMR spectra (solvent: DMSO) and B) a comparison of the experimentally obtained spectrum (positive ion mode) and simulated isotopic patterns of BAU. Experimental *m*/*z* 1169.4689 [*M*+H]^+^ matches the theoretical value of 1169.4685.

**Figure 3:**
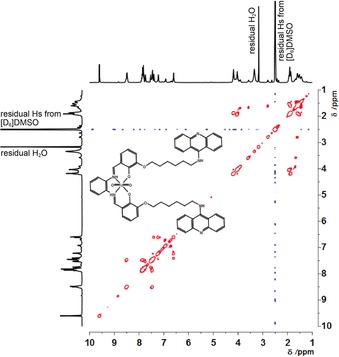
2D COSY NMR spectrum (400 MHz, [D_6_]DMSO) of BAU.

NMR spectroscopy and MS analyses confirmed the structure of BAU, and validated our synthetic scheme. The ^1^H and ^13^C chemical shifts of BAU were assigned upon standard 1D and 2D NMR spectroscopy characterization (Figures 2 and 3). Specifically, the 2D NMR spectroscopy measurements (Figure 3) allowed the spin systems to be assigned to the following structural patterns: the salophen–UO_2_ complex, the acridine moiety, and the connecting hexyl spacer. In particular, the signal of the magnetic resonances attributable to the azomethine groups of the salophen–UO_2_ complex was observed at *δ*=9.60 ppm, whereas significant magnetic resonances for the three phenyl rings bridged by these azomethine groups appeared in the range of *δ*=7.22–6.56 ppm. The signals corresponding to the aromatic pattern of the acridine moiety were assigned in the range of *δ*=8.45–7.30 ppm. The presence of the hexyl chain spacer was confirmed by the magnetic resonances of the CH_2_ groups attached to the phenyl group of the salophen–UO_2_ complex and the amino group at the 9‐position of acridine, which were underpinned at *δ*=4.15 and 3.93 ppm, respectively. Figures S1–S5 in the Supporting Information allow for further ^1^H and ^13^C NMR characterization of the intermediate synthesized compounds (i.e., compounds **3**–**7**). The complementary characterization of the exact mass by means of HRMS (ESI) confirmed the formation of target BAU (Figure 2 B). Based on the thorough structural confirmation by means of NMR spectroscopy and ESI‐MS, we thus explicitly showed the feasibility of synthesizing a metallointercalator molecule associating a very heavy element, such as uranium.

**Figure 4:**
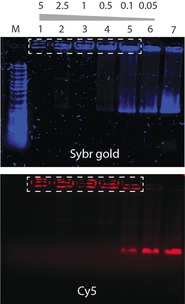
Gel electrophoresis reveals binding of a BAU intercalator to DNA origami nanostructures. The origami nanoplates were labeled with Cy5 fluorophores (red) and incubated with different molar ratios of intercalator per DNA base pair, ranging from 5 to 0.05 in lanes 1 to 6, respectively. Notably, the SYBR Gold staining intensity decreases in the presence of high amounts of BAU (lanes 1–2) and the electrophoretic mobility of the origami plates is significantly altered upon binding of the positively charged intercalator (lanes 1–4) relative to the free origami control (lane 7). This is a 0.7 % agarose gel run at 7 V cm^−1^ in TBE‐Mg buffer for 130 min at 4 °C. M: GeneRuler DNA Ladder Mix (SM0333, ThermoFisher).

As a prerequisite to use BAU as a DNA electron dye, it should bind strongly to DNA. We performed gel electrophoresis to evaluate the bulk binding properties of BAU to DNA origami nanostructures (Figure 4). DNA origami nanostructures, containing fluorescent Cy5‐labeled staple strands, were incubated with variable molar ratios of BAU to DNA base pairs, starting from an excess value of 5 BAU bp^−1^ to lower ratios down to 0.05 BAU bp^−1^, as calculated from the scaffold length of the DNA origami. Visualization of the origami nanostructures was achieved by fluorescent imaging of the Cy5 fluorophores, as well as by staining with SYBR Gold. Figure 4 shows the data and elucidates two important points: 1) A close look at the immobile band indicated inside the dashed white rectangles reveals an increasing intensity in the SYBR Gold channel with lower BAU concentration, but a uniform intensity in the Cy5 channel. This suggests that BAU competitively inhibits the binding of SYBR Gold to DNA nanoplates, most likely through intercalation. Our observation is consistent with the general consensus that bis‐acridine moieties are indeed excellent intercalators.^15^, ^16^ 2) BAU binding alters the electrophoretic mobility of the DNA origami, since we observe that the origami structures become completely immobilized inside the gel pockets (lanes 1 to 4), and can only acquire partial mobility below stoichiometric ratios of 0.5 BAU bp^−1^ (lanes 5 and 6). This is attributed to both the positive charge of BAU and interorigami crosslinking that can occur if the two acridine intercalating units of BAU bind to two different origami plates. Indeed, TEM images taken from these complexes confirmed such BAU‐induced origami interactions (Figure S6). Collectively, the results of electrophoretic analysis indicate that BAU binds tightly to DNA, thereby suggesting its potential to be used as a DNA electron dye.

### TEM imaging

**Figure 5:**
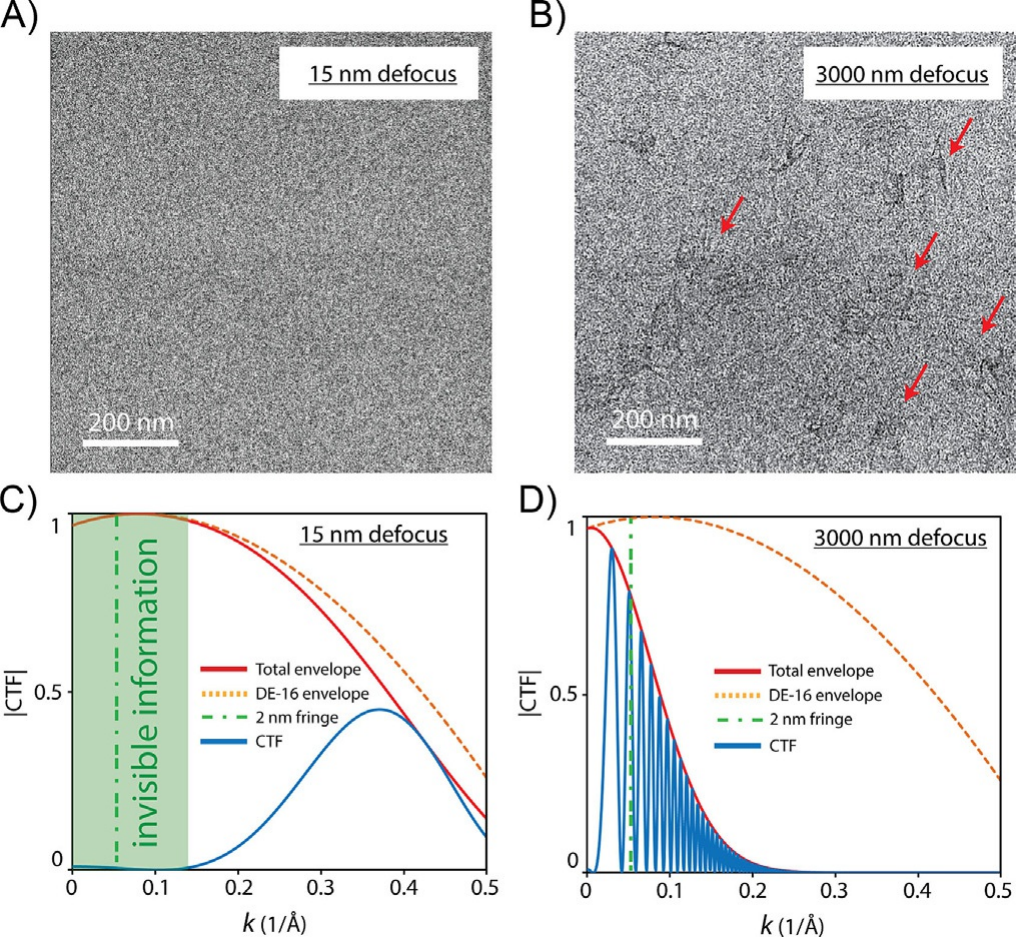
Conventional wide‐field TEM suffers from poor contrast for imaging of stained DNA origami nanostructures. A), B) Wide‐field TEM images of DNA origami stained with UA, taken by a DDD detector (DE‐16) assisted by dose fractionation and drift correction. The origami plates are only partially visible in (B), but completely invisible under near‐focus conditions in (A). Contrast transfer function (**⋅**) simulations for our aberration‐corrected Titan microscope at different objective‐lens defocus values of C) 15 and D) 3000 nm; **—**: total envelope, **⋅⋅⋅⋅**: DE‐16 envelope, **–⋅–**: 2 nm fringe, **—**: CTF. The simulation input values were set according to our microscope settings.

We find that it is not possible to image the stained DNA nanostructures with high contrast in wide‐field TEM, even with a state‐of‐the‐art direct detection device (DDD). In recent years, the field of TEM has gone through a dramatic improvement in instrumentation, especially due to the emergence of DDD technology. By eliminating the scintillator layer, the DDDs became advantageous over conventional charge‐coupled device (CCD) cameras because they yield a higher dynamic range, higher detective quantum efficiency for all spatial frequencies up to the Nyquist limit, sample‐motion correction, and a lower shot noise.^17^ Because improvements in the detector would be beneficial to boost the contrast in wide‐field TEM, a recent state‐of‐the‐art DDD (Model: DE16, Direct Electron, California, USA) was employed in our aberration‐corrected microscope. Figure 5 A and B shows the best micrographs that we could acquire under near‐focus and strongly defocused objective‐lens settings, respectively. It is seen that the UA‐stained DNA structures are completely invisible at near focus (Figure 5 A), but they do appear very faintly under the strongly defocused illumination conditions (Figure 5 B). Only the origami main rectangle and occasionally the side arms (4 nm wide) are distinguishable, but the dsDNA loop at the bottom is not recognized at all. Note that the contrast in Figure 5 A and B is very faint because we do not perform negative staining, with which the contrast is generated by a shadow image of the DNA in a uniform stain background. Rather, to investigate the “selectivity” of electron dyes, the contrast is generated by direct interaction of the compounds with DNA (that is, positive staining). To investigate the visibility at the single‐molecule level, our origami contains only one layer of DNA, unlike multilayer 3D DNA origami designs,^18^ and hence, it yields very low electron scattering.

The poor visibility of the stained DNA origami structures in wide‐field TEM imaging is explained by electron–optical reasons. For materials science specimens (mostly metals and ceramics), wide‐field TEM at a focus close to zero is extensively used to resolve microscopic features with atomic resolution (even below 1 Å resolution). However, in the case of biological samples, TEM imaging at near‐focus conditions fails to provide enough contrast for visualization.^19^ This is illustrated in Figure 5 C and D: we calculated the CTF of our aberration‐corrected Titan microscope at near‐focus and at strongly defocused imaging conditions, respectively. The CTF is a mathematical function that takes into account various imaging parameters, such as the objective‐lens defocus, acceleration voltage, and aberration coefficients, and it plots the phase‐to‐amplitude conversion efficiency as a function of spatial frequencies of the electron wave (i.e., the *k* vectors). For example, in the case of near‐focus imaging (Figure 5 C), we observe no information transfer for the 2 nm fringe, which is the periodicity of the stacked DNA bundles within the origami rectangle, although the microscope can still resolve spatial features up to 2 Å (the right side of the curve with *k*=0.5 Å^−1^). All spatial frequencies in the green‐highlighted area in Figure 5 C will be absent in the final image. If the objective lens is strongly defocused up to 3000 nm (Figure 5 D), however, the 2 nm phase component is converted to amplitude with better efficiency, although damped by the total spatial and temporal envelope. At this strong‐defocus illumination, the CTF oscillates rapidly, with its first zero shifting to lower spatial frequencies, which consequently results in a loss of resolution (well below 5 Å, that is, more than a 50 % drop in resolution relative to Figure 5 C). We therefore conclude that wide‐field TEM is not a good approach to visualize DNA nanostructures with high contrast.

**Figure 6:**
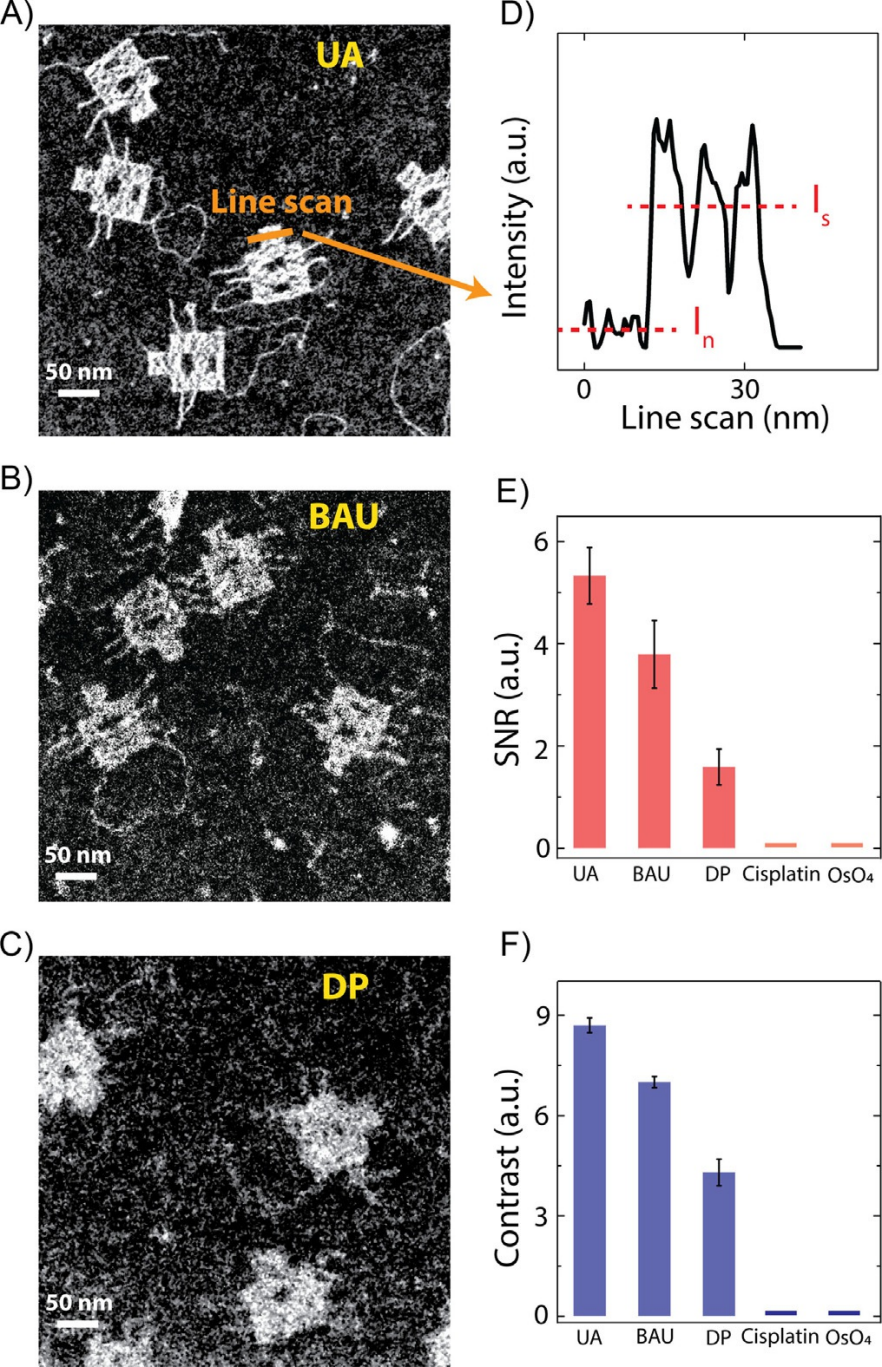
STEM imaging visualized stained DNA nanostructures at the single‐molecule level. STEM images of DNA origami nanoplates stained with UA (A), BAU (B), and DP (C); see the Experimental Section for the dye concentrations. All images are single‐frame acquisitions, without any class averaging. D) High‐angle annular dark‐field (HAADF) signal taken along the dashed line in A). Statistical analysis of the SNR (E) and contrast (F) obtained for each dye. The processed HAADF signals were extracted from different origami plates, similar to the example shown in D).

Unlike wide‐field TEM, we find that the STEM technique is suited to probe the DNA–dye interactions. STEM is an imaging technique with a very different image‐formation mechanism,^20^ which does not suffer from the CTF constraints. One main advantage of STEM over TEM for our purpose hinges on the fact that the obtained contrast is almost quadratically proportional to the atomic number (*Z*‐contrast imaging),^20^ and dyes containing elements with atomic numbers of 92 (UA, BAU) or 78 (DP) thus generate a crisp contrast over the substrate with a low atomic number of 6 (carbon). Figure 6 A–C summarizes our STEM results for electron dyes that rendered origami nanoplates visible. All images were taken under the same STEM acquisition parameters in the microscope and were treated with a similar despeckle noise reduction and contrast adjustment for better visibility.

Importantly, our new BAU dye is seen to yield good DNA contrast in STEM (Figure 6 B). For BAU and DP, we see contrast enhancement not only for the main origami rectangle (72 nm×50 nm), but also for individual DNA strands (the floppy loop at the bottom that is only 2 nm (single DNA helix) wide, as well as side arms that are composed of 2 DNA helices, i.e., 4 nm wide). Such high‐contrast single‐shot STEM images of DNA supported on commercial carbon membranes indicate that STEM is a particularly fit technique for imaging stained DNA nanostructures. Excitingly, unlike staining protocols used for tissue sections, it is clear that no post‐fixation process is necessary for our DNA origami samples, which presents an important advantage for artefact‐free imaging. To probe whether it was possible to increase the contrast even further, we stained the DNA nanoplates with high stain concentrations and increased the incubation times. We found that the contrast saturated at a certain concentration for each dye (Figure S7). The images in Figure 6 thus represent the highest contrast that could be achieved for each compound; likely representing the maximum number of dye molecules bound to the DNA strands.

We also investigated other staining agents reported in the literature, such as OsO_4_ and cisplatin, but we found no visibility in STEM imaging of DNA origami with these compounds. OsO_4_, with its high atomic number of 76 for osmium, plays a unique role in TEM imaging of biological samples,^21^ and it is often reported as a fixative agent for plastic‐embedded tissue sections. Bahr reported that OsO_4_ did not react with nucleic acids,^21^ although no microscopic evidence was presented. To attempt to provide microscopic evidence for DNA binding, we stained our origami nanoplates with OsO_4_ and performed STEM imaging. We could, however, not detect any discernible contrast. Cisplatin, one of the most successful antitumor drugs, can potentially also be considered as an electron dye. It is known that cisplatin binds quasi‐covalently to DNA on the N^7^‐positions of the bases, which consequently causes double‐helix unwinding, kinking, and DNA crosslinking.^2^, ^3^, ^22^, ^23^, ^24^ Despite this purported covalent affinity towards DNA, we were unable to visualize cisplatin‐stained DNA origami through STEM. Some studies pointed out that cisplatin required long incubation times to interact with DNA.^3^, ^7^, ^23^ We therefore prolonged the incubation time of cisplatin with DNA origami nanoplates on the TEM grid up to 2 days. After this long incubation period, we could observe large amounts of nanoparticles deposited on the TEM grids, but unfortunately still not any visible DNA origami (Figure S8). Our microscopic findings thus show that cisplatin and OsO_4_ are not suitable for in vitro staining studies of DNA.

We quantified the contrast of the stained DNA origami nanoplates. Different origami nanoplates were selected from random acquisitions at different locations on the TEM grids, and the HAADF detector signals were extracted along the origami main body (Figure 6 D). SNR and contrast were computed according to SNR=(*I*_s_−*I*_n_)/Std_n_ and contrast=(*I*_s_−*I*_n_)/*I*_n_, respectively, in which *I*_s_ denotes the average signal along the origami main rectangle, *I*_n_ is the average background noise measured along the carbon support, and Std_n_ is the standard deviation of the background noise. Remarkably, Figure 6 E and F shows that BAU, with the bis‐acridine intercalating ligand, provides an excellent SNR and contrast, approaching the values of the UA, but now as a positive‐stain intercalator. The SNR and contrast values for BAU exceed that of DP, with the terpyridine intercalating moiety (more than twice as high SNR and 60 % higher contrast). This indicates that our design strategy to consider the bis‐acridine ligand was indeed more effective than the intensively studied terpyridine conjugation. Notably, although the backbone binder UA provides the highest contrast among the stains, we aimed to synthesize and investigate intercalating electron dyes. When using the intercalating BAU and DP compounds, we always observed a higher noise (carbon appears to be brighter), which we could not avoid, even after several washing steps. This may be due to some affinity of the intercalating ligands to the surface of amorphous carbon. Nevertheless, single‐DNA molecules are clearly visible over the background stained with BAU and DP intercalating dyes.

## Discussion

We designed a new intercalating electron dye and presented single‐molecule visualizations of DNA. In our experiments, *Z*‐contrast STEM images were generated through direct interaction of these dyes with the DNA strands, instead of conventional negative staining protocols in which the contrast is rendered through a shadow image of DNA. Table 1 provides an overview of association constants, number of dye molecules bound to each DNA base pair, and the electron‐scattering capacity of the heavy‐metal center. Various of these attributes are important for rendering high‐contrast images, as discussed below.

**Table 1:** Biochemistry of DNA–dye interactions.

	UA	BAU	DP
binding mechanism	backbone	intercalation	intercalation^[a]^
*K*_a_ [m ^−1^]	8×10^6[1]^	1.2×10^4^	1.3×10^5^– 101.2×10^6[30, 31]^
no. bound dyes per	1	0.25^37^ (ToTo	0.2^30^, ^31^
base pair		bis‐intercalator)	
*Z* of the core metal^[b]^	92	92	78

[a] Eventually becoming covalent after ligand exchange. [b] *Z*: atomic number.

First, we note that the common denominator between all of these dyes is their high binding constants, in the range of 10^4^–10^6^ m ^−1^. This strong affinity is indispensable for visualization, rendering single‐molecule DNA visible even after several washing steps during sample preparation. Regarding BAU, this strong binding affinity was verified by means of gel electrophoresis (Figure 4). The use of the bis‐acridine moiety as the intercalating ligand in the BAU design approach was intentional, with respect to a high binding constant, because it was shown that bis‐acridines had a substantially higher affinity to DNA than that of their corresponding mono‐acridine analogues.^15^, ^16^ Next to a high DNA affinity, another advantage of bis‐acridine conjugation to the salophen ligand containing uranium is that bis‐acridine moieties are known to be biocompatible agents with excellent antitumor properties.^15^, ^16^

Second, the dye density along the DNA helix should be maximized to obtain the highest scattering efficiency. Certainly, UA provides the highest value of one dye bound per base pair, compared with BAU and DP that perform a factor of 4 or 5 worse than UA. Beer and Moudrianakisen showed that attachment of three heavy markers per base pair was required for one full amplitude contrast onto thin carbon membranes in wide‐field TEM,^25^ whereas staining with UA could yield a maximum of only one heavy atom of uranium. This, together with phase‐contrast limitations imposed by CTFs shown in Figure 5 C and D, explains the faint contrast that one obtains in normal wide‐field TEM mode, for which even utilization of a DDD is not helpful to boost the contrast (Figure 5 A and B).

Finally, although on the basis of theoretical arguments, the STEM contrast is expected to scale as approximately *Z* ^2^ versus atomic number—technically the current in the HAADF detector reaches a plateau—which consequently saturates the grayscale values in the STEM images and impedes heavy elements from being distinguishable from one another. For example, Ferrer et al. showed that the intensity difference between the Au (*Z*=79) and Pd (*Z*=46) columns in a Au/Pd nanoparticle was very small.^26^ Accordingly, we expect that the obtained STEM contrast for the dyes mentioned in Table 1 (with *Z*=92 for U and *Z*=78 for Pt) is not likely to be strongly dependent on the core heavy element. In other words, we estimate that the effects of binding constant and dye loading density are much more pronounced in determining the overall contrast in STEM than the atomic number of the core heavy metal. Note that this statement is only true for heavy elements, whereas *Z*‐contrast imaging remains strongly beneficial if heavy atoms are supported on light substrates, such as carbon (Figure 6).

Maximizing the above‐mentioned parameters provides a strategy for biochemists to further develop highly efficient electron dyes. Most importantly, the binding constant and dye‐loading density significantly contribute to contrast. We have shown the feasibility of bis‐acridine conjugation to the salophen ring as one strategy for incorporating uranium (in BAU). Also, the Pt^II^ compounds containing terpyridine moieties (in DP) are shown to be suitable candidates as intercalating electron dyes. The application of many platinum(II) or osmium complexes have been demonstrated for chromatin imaging. In fact, there are numerous papers presenting such chromatin data,^4^, ^7^, ^27^ but virtually none on in vitro imaging of single‐molecule DNA through intercalation binding, which is the focus of our work. Based on our quantitative analyses in Figure 6 E and F, such compounds perform more poorly than our bis‐acridine design strategy for single‐molecule visualization because our BAU compound has two DNA binding sites and a spatially distant heavy metal, which allows a tweezer‐like binding to the DNA molecule. Tethering other intercalating ligands can be considered. The rich coordination chemistry of the transition metals, together with the diverse conjugation possibilities to flat aromatic ligands, are promising factors for the future synthesis of electron dyes. We hope that our findings and methodology will spur the interests of chemists to further develop highly efficient intercalating electron dyes.

## Conclusion

We imaged single DNA molecules with high contrast by using a newly designed intercalating molecule, and we compared the results to those for other electron dyes. By introducing DNA origami as a microscopy test object and by selecting STEM over conventional wide‐field TEM, we could characterize electron dyes that were appropriate for single‐molecule DNA visualization. Imaging artefacts in previous investigations were eliminated, as a result of facile sample preparation and the absence of other biological molecules, such as lipids and proteins. Our methodology is suited for investigation of other newly synthesized electron dyes in the future. Looking forward, one intriguing application of intercalating electron dyes is in multicolor electron microscopy of biological systems. The first ever multicolor TEM images of such samples was shown by Adams et al. in 2016, who visualized different cellular components, very similar to what multicolor fluorescence microscopy could offer, but with the full spatial resolution of TEM.^28^ This is opening up new possibilities to push the resolution limits in life‐science TEM, and thus, expanding our understanding of the molecular processes of life.

## Experimental Section

**Staining compounds**: The BAU dye was synthetized as shown in Scheme 1. To obtain an optimal linker length between the DNA intercalating ligand (i.e., the acridine moiety) and the TEM‐contrast‐enhancer scaffold (the uranyl unit), commercially available 9‐chloroacridine (**1**) was reacted with 6‐aminohexan‐1‐ol (**2**), as described previously.^29^ The isolated 9‐hexylaminoacridine compound (**3**) was tosylated to afford **4**, which was subsequently reacted with the selective allyl‐protected 2‐(2‐propenyloxy)‐3‐hydroxybenzaldehyde (**5**) to yield compound **6**, with a benzaldehyde unit. The successful deprotection of **6** provided the free hydroxybenzaldehyde unit (**7**). The salophen base unit was synthesized by heating **7** at reflux with 1,2‐benzenediamine (**8**) in methanol, followed by treating the mixture with UA dihydrate to afford the uranyl–bis‐acridine metallointercalator (**9**). For details of the complete synthesis, please see the Supporting Information. The stock concentration for staining experiments was 250 μm in DMSO. For staining compounds as reference measurements, we selected compounds with a known chemistry and specific DNA binding modes; commercial drug cocktails of unknown types of interactions with DNA were neglected. For UA, a solution of 2 % UA in Milli‐Q water was used, which was filtered through a 0.2 μm polytetrafluoroethylene (PTFE) membrane. Among the huge family of Pt^II^ compounds, the rationale for choosing an appropriate molecule was to have the simplest molecule with the presence of a common intercalating motif, namely, the planar terpyridine subunit. Based on this, we selected DP (Sigma Aldrich; 10 mm in TE buffer).^30^, ^31^, ^32^

**NMR spectroscopy**: NMR (^1^H and ^13^C) spectroscopy measurements were performed by using either a Bruker Avance III 400 spectrometer (^1^H, 400 MHz; ^13^C, 100 MHz) or a Bruker Avance 500 MHz spectrometer equipped with Ultrashield magnets (^1^H, 500 MHz; ^13^C, 125 MHz). The residual solvent signals were employed for shift correction (for ^1^H NMR spectra at *δ*=2.50, 4.87, and 7.26 ppm, for ^13^C NMR at *δ*=39.52, 49.00, and 77.16 ppm, respectively, for the following deuterated solvents CD_3_OD, [D_6_]DMSO, and CDCl_3_). Multiplets (m) are reported over the range where they appeared at the indicated field strength.

**MS analysis**: Fast atom bombardment (FAB) and HRMS results were measured with a MAT95 (Finnigan) mass spectrometer. ESI‐MS data were recorded on a Q‐Exactive (Orbitrap) mass spectrometer (Thermo Fisher Scientific, San Jose, CA, USA) equipped with a HESI II probe. The instrument was calibrated over the *m*/*z* range of 74–1822 by using premixed calibration solutions (Thermo Scientific). A constant spray voltage of 4.7 kV and a dimensionless sheath gas of 5 were applied. The capillary temperature and the S‐lens RF level were set to 320 °C and 62.0 V, respectively. The samples were dissolved to a concentration of 0.05 mg mL^−1^ in a mixture of THF/MeOH (3:2) containing 100 μmol sodium trifluoroacetate (NaTFA) and infused with a flow of 5 mL min^−1^.

**Association constant (*K***_**a**_ **) measurements of BAU**: Quantification of the association constant of BAU toward dsDNA was performed by means of UV/Vis absorbance titration following two different procedures. In the first set of experiments, a freshly prepared solution of BAU (45 μm in 11.5 % DMSO and 88.5 % TE‐Mg buffer (20 mm Tris, 2 mm ethylenediaminetetraacetic acid (EDTA), 12.5 mm MgCl_2_, pH 8)) was transferred into a quartz cuvette. Subsequently, small volumes (2–10 μL) of a solution of dsDNA of known concentration were subsequently added to the solution of ligand and incubated for 5 min, followed by recording of the UV/Vis spectra. Dilution of the ligand was taken into account during data analysis. In the second set of experiments, a freshly prepared solution of BAU (40 μm in 10 % DMSO and 90 % TE‐Mg buffer) was divided into different aliquots and individually mixed with a known amount of dsDNA and incubated for 5 min, followed by recording of the UV/Vis spectra. In each sample, the volume was maintained constant (280 μL). All UV/Vis absorbance spectra were recorded by using a Cary Series UV/Vis spectrophotometer, Agilent Technologies. For these experiments, a 5438 bp bacterial plasmid (109Z5^33^) was used as dsDNA. The concentration of dsDNA stock solution (expressed as bp concentration) was determined by means of UV/Vis measurements in TE buffer (20 mm Tris, 2 mm EDTA pH 8). The affinity constants were determined from changes in the absorbance, according to a reported equation,^34^ which was derived from the previously reported neighbor‐exclusion model.^35^

**DNA origami as a calibration tool for TEM imaging**: We designed a 2D DNA origami nanoplate as a test object for the scattering contrast of dsDNA (Figure 1). Our origami structure contained various DNA features of interests, such as side arms (4 nm wide, 27 and 43 nm long), two cavities in the middle (4 and 8 nm wide, 19 nm long), and a (flexible) individual dsDNA (2 nm wide) molecule loop at the bottom. Incorporation of these features into the design made it convenient to evaluate if various contrast agents could visualize individual DNA molecules and more extended DNA structures. For details of the origami design and its characterization, we refer to our previous work.^36^

**TEM sample preparation**: Commercial carbon‐coated TEM grids (nominal 3–4 nm thin carbon supported by a 5–6 nm formvar layer; Electron Microscopy Science, USA) were used to support the origami nanoplates. The origami solution (4 μL, 5 nm) was applied to freshly glow‐discharged TEM grids and incubated for 2 min. Grids were washed with ultraclean Milli‐Q water to remove unadhered origami plates. Next, without drying the sample, the staining compound was pipetted onto the TEM grids and left to react for 1 min. Finally, the grids were thoroughly washed to remove all chemicals from the carbon surface. Removing the residual stain in the last step is a key point to avoid generation of contrast by the common negative staining protocols (for which the thickness variation of the stain layer across the biomolecule creates detectable mass‐thickness contrast). In the current work, the observed contrast was instead the consequence of direct physiochemical interaction of the staining chemicals with DNA. Following the above TEM preparation protocol, we obtained uniform distributions of origami nanoplates with a high density on the TEM grids, which allowed facile investigation of the staining compounds; this is a clear advantage over the tedious sample preparation protocols for tissue sections or other DNA‐containing biological samples.

**TEM imaging**: For S/TEM imaging, we used a FEI Titan microscope equipped with a postspecimen aberration corrector operating at an acceleration voltage of 300 kV. Imaging at 300 kV was preferred over 80 kV for lower ionization damage to DNA. A HAADF detector (Fischione, USA) at a camera length of 28.3 cm was used for obtaining mass‐thickness‐dominated contrast (*Z*‐contrast). For quantitative comparison, all STEM images were acquired with the same imaging parameters (convergence angle, dwell time, pixel size) and postprocessing for contrast evaluation. In TEM mode, the third‐order spherical aberration (C3) coefficient was set to zero in the image corrector to minimize information delocalization.

## Conflict of interest

*The authors declare no conflict of interest*.

## Supporting information

As a service to our authors and readers, this journal provides supporting information supplied by the authors. Such materials are peer reviewed and may be re‐organized for online delivery, but are not copy‐edited or typeset. Technical support issues arising from supporting information (other than missing files) should be addressed to the authors.

Supplementary
