# Supplementary material for: Intercalating Electron Dyes for TEM Visualization of DNA at the Single‐Molecule Level
Source: Chembiochem. 2019 Feb 7;20(6):822–30. doi: 10.1002/cbic.201800638 (PMC6470888; doi:10.1002/cbic.201800638)
Supplement: Supplementary file 1 — Supplementary [file CBIC-20-822-s001.pdf]

CHEMBIOCHEM

Supporting Information

Intercalating Electron Dyes for TEM Visualization of DNA at the Single-Molecule Level

Yoones Kabiri,[a] Alessandro Angelin,[b] Ishtiaq Ahmed,[b] Hatice Mutlu,[c, d] Jens Bauer,[b] Christof M. Niemeyer,[b] Henny Zandbergen,*[a] and Cees Dekker*[a]

cbic_201800638_sm_miscellaneous_information.pdf

Copyright WILEY-VCH Verlag GmbH & Co. KGaA, 69469 Weinheim, Germany, 2016.

Contents:

1. Synthesis of bis-acridine-uranyl (BAU) metallo-intercalator, and NMR spectra of intermediate compounds

2. Cross-linking of DNA origami via BAU tethering

3. The effects of dye concentration and incubation time on the contrast of the stained DNA origami nanoplates

4. Nanoparticle deposition onto TEM grids after incubation with cisplatin

1. Synthesis of bis-acridine-uranyl (BAU) metallo-intercalator, and NMR spectra of intermediate compounds

Uranyl metallo-intercalator based on the Salophen base unit was synthesized by starting with commercially available 9-chloroacridine. In the first step, a spacer chain was attached by treating 9-chloroacridine (**1**) with 6-aminohexan-1-ol (**2**) followed by tosylation to get the tosylated product (**4**). Selective allyl protected 2-(2-Propenyloxy)-3-hydroxybenzaldehyde (**5**), obtained according to the literature, [1] was refluxed with tosyl derivative (**4**) to afford compound **6** having benzaldehyde attached with an acridine unit through spacer chain, which on deprotection gave the free hydroxybenzaldehyde unit (**7**). The salophen base unit was synthesized by refluxing compound **7** with 1,2-benzenediamine (**8**) in methanol followed by treating the mixture with uranyl acetate dihydrate to afford uranyl-bis-acridine metallo-intercalator (**9**).

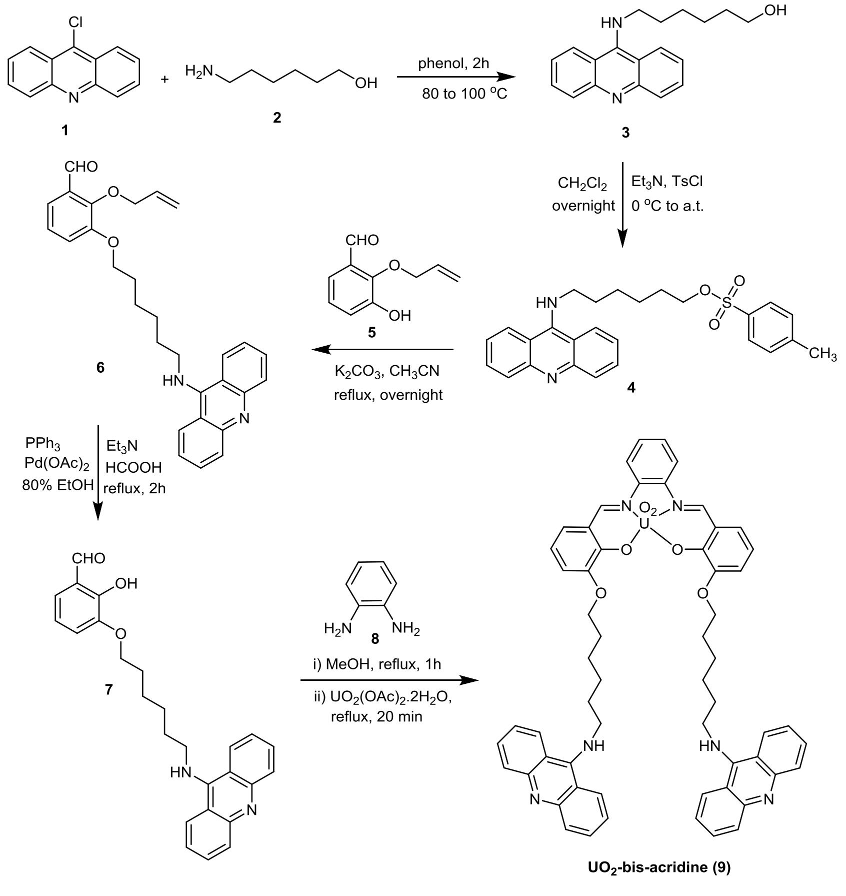

Scheme 1: Synthesis of uranyl-bis-acridine metallo-intercalator (**9**)

6-(acridin-9-ylamino)hexan-1-ol (compound 3)

A mixture of 9-chloroacridine (**1**) (3.12 g, 14.6 mmol, 1.0 eq) and 6-aminohexan-1-ol (**2**) (1.71 g, 14.6 mmol, 1.0 eq) was added to phenol (20 g, at 80 °C), and the resulting mixture was heated for 2 h at 100 ^{o}C. The reaction mixture was cooled to room temperature, and the solvent was removed under reduced pressure at 70°C. The brown residue was refluxed with ethanol and further treated with minute amounts of diethylether to afford crude product **3** (93 %, 4 g, 13.6 mmol) as a yellow solid.

^{1}H NMR (500 MHz, CD_3OD): δ (ppm) 1.36-140 (m, 2H), 1.49-1.61 (m, 6H), 3.22 (t,*J* = 7.2Hz, 2H), 4.11 (*J* = 7.7, 2H), 7.48-7.55 (m, 2H), 7.79 (d, *J* = 8.6Hz, 2H), 7.88-7.99 (m, 2H), 8.04, s, 1H), 8.45 (d, *J* = 8.6 Hz, 2H).

^{13}C NMR (125 MHz, CD_3OD): δ (ppm) 26.2, 28.9, 29.2, 32.1, 49.1, 61.4, 118.2, 123.5, 125.2, 127.7, 130.9, 134.9, 135.5, 162.3.

HRMS-FAB: theoretical $C_{19}H_{23}N_2O$ [M+H]$^{+}$ 295.1859, experimental 295.1856

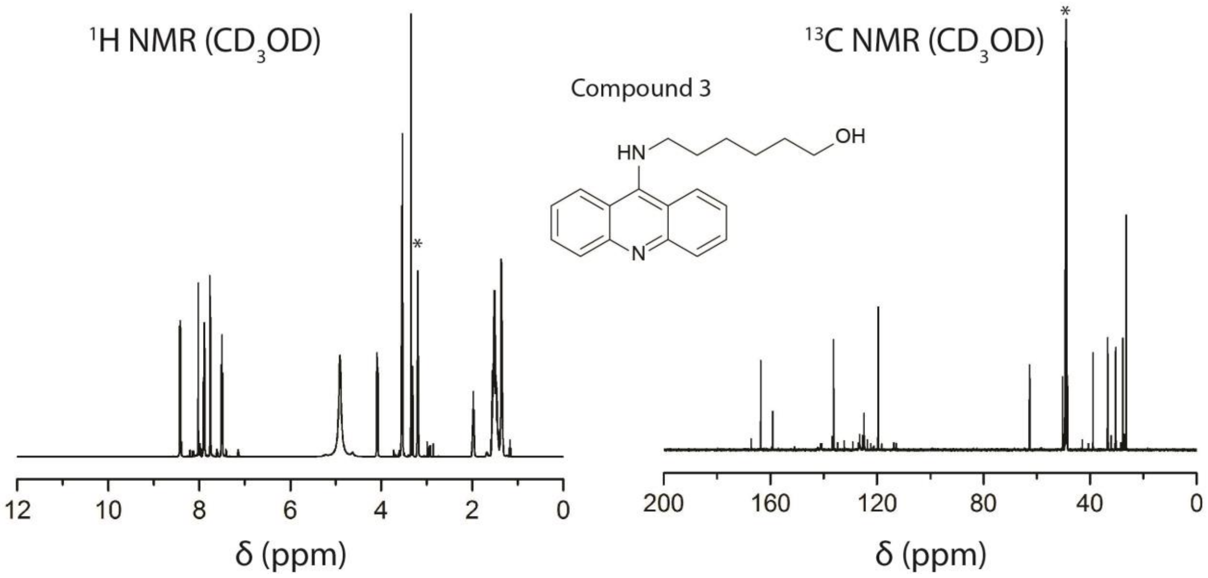

Figure S1: ^{1}H (500 MHz, CD_3OD) and ^{13}C (125 MHz, CD_3OD) NMR spectra of compound 3

6-(acridin-9-yl-amino)hexyl-4-methylbenzenesulfonate (compound 4)

Triethylamine (1.13 mL, 8.10 mmol, 1.3 eq) was added to a mixture of 6-(acridin-9-ylamino)hexan-1-ol (**3**) (1.83 g, 6.23mmol, 1 eq) in anhydrous dichloromethane (20 mL) under inert atmosphere. The mixture was stirred for 1 hour at ambient temperature. The solution of *p*-toluenesulfonyl chloride (1.43 g, 7.48mmol, 1.2 eq) in 10 mL of anhydrous dichloromethane) was then added in a dropwise manner to the reaction mixture at 0 ^{o}C. The reaction mixture was stirred overnight at ambient temperature; subsequently dichloromethane (100 mL) was added to quench the reaction. The organic phase was washed with water (3 x

100 mL and brine (1 x 100 mL), and afterwards dried over anhydrous $MgSO_4$. The solvent was then removed under reduced pressure to afford the crude tosylated product **4** (97 %, 2.71 g, 6.04mmol) as a yellow solid.

^{1}H NMR (500 MHz, $CDCl_3$): δ (ppm) 1.34-147 (m, 4H), 1.55-1.72(m, 2H), 1.91-2.02 (m, 2H), 2.45 (s, 3H), 3.99-4.09 (m, 4H), 7.06-7.12 (m, 2H), 7.31-7.35 (m, 4H), 7.77 (d, *J* = 8.2 Hz, 2H), 8.02 (d, *J* = 8.2 Hz, 2H), 8.17 (d, *J* = 8.6 Hz, 2H).

^{13}C NMR (125 MHz, $CDCl_3$): δ (ppm) 21.6, 25.0, 26.2, 28.6, 29.9, 53.4, 70.4, 119.9, 122.9, 124.8, 126.0, 127.8, 129.0, 129.8, 133.0, 133.5, 140.3, 142.6, 144.7, 156.2

HRMS-FAB: theoretical $C_{26}H_{29}N_2O_3S$ $[M+H]^+$ 449.1730 experimental 449.1739

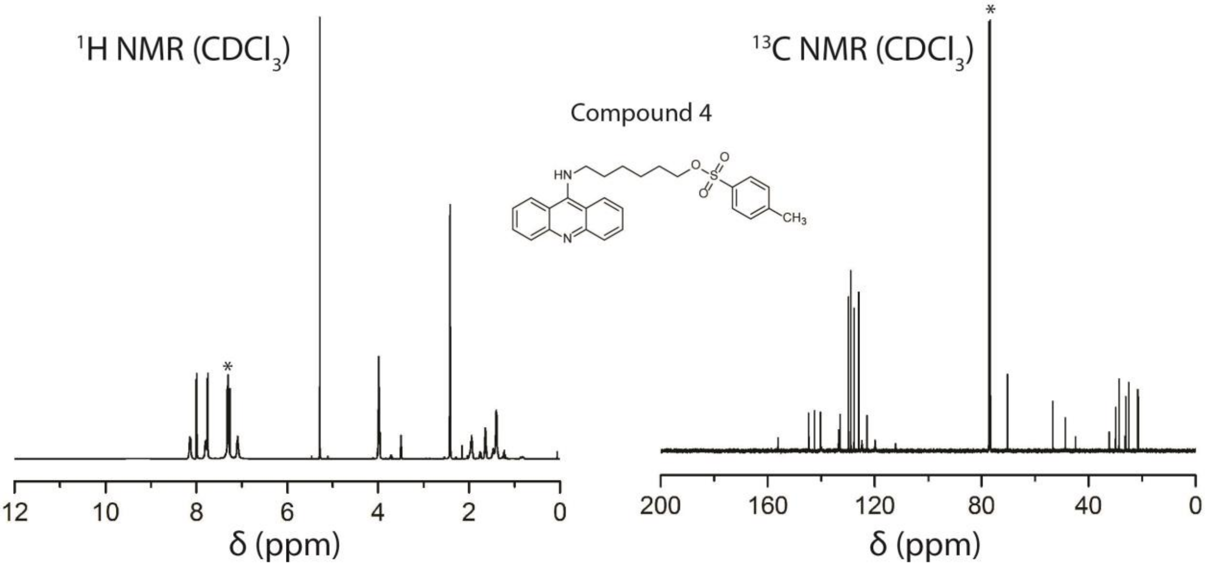

Figure S2: ^{1}H (500 MHz, $CDCl_3$) and ^{13}C (125 MHz, $CDCl_3$) NMR spectra of compound 4

2-(2-Propenyloxy)-3-hydroxybenzaldehyde (compound 5)

Selective protection of 2,3-dihydroxybenzaldehyde was done according to the literature procedure [1]. Sodium hydride (1.15 g, 0.038 mol, 1.05 eq), which was washed with petroleum ether 40-60 ^{o}C before use, was added to the mixture of 2,3-dihydroxybenzaldehyde (5.0 g, 0.036 mol, 1.0 eq) in DMSO (65 mL). After the mixture was stirred at ambient temperature for 2 h, 3-bromo-1-propene (4.5 g, 0.038 mol, 1.05 eq) was added in a dropwise fashion. Subsequently, the reaction mixture was stirred at ambient temperature for 24 h. The product was poured into water (200 mL) and extracted with chloroform (3 x 150 mL). The combined organic phase was washed with distilled water (3 x 150 mL), dried over $MgSO_4$ and concentrated under reduced pressure. The residue was purified by silica gel column chromatography (chloroform was used as eluent), followed by recrystallization (petroleum ether 40-60 ^{o}C /ether 95/5) to afford pure the product **5** (65 %, 4.20 g, 0.024 mol) as white needles.

^{1}H NMR (500 MHz, $CDCl_3$): δ (ppm) 4.58-4.62 (m, 2H), 5.34-5.46 (m, 2H), 5.98 (bs, 1H), 6.07-6.17 (m, 1H), 7.13-7.23 (m, 2H), 7.39 (dd, *J* = 7.7 Hz and 1.7 Hz, 1H), 10.28 (s, 1H).

^{13}C NMR (125 MHz, $CDCl_3$): δ (ppm) 77.3, 120.1, 121.5, 121.8, 125.1, 129.3, 132.4, 147.8, 149.7, 189.8.

HRMS-FAB: theoretical $C_{10}H_{11}O_3$ $[M+H]^+$ 179.1625, experimental 179.1621

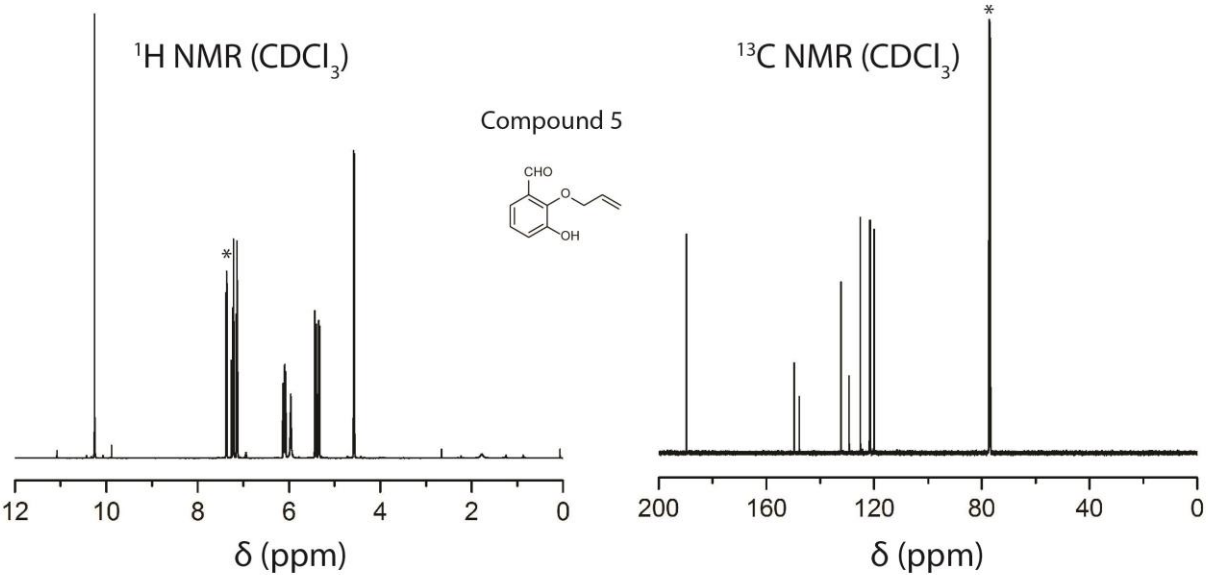

Figure S3: ^{1}H (500 MHz, $CDCl_3$) and ^{13}C (125 MHz, $CDCl_3$) of compound 5

3-((6-(acridin-9-ylamino)hexyl)oxy)-2-(allyloxy)benzaldehyde (compound 6)

To a suspension of 6-(acridin-9-ylamino)hexyl-4-methylbenzenesulfonate (**4**) (2.71 g, 6.04 mmol, 1.0 eq) and 2-(2-Propenyloxy)-3-hydroxybenzaldehyde (5) (1.07 g, 6.00 mmol, 1.0 eq) in anhydrous acetonitrile (200 mL), potassium carbonate (2.60 g, 18.7 mmol, 3.12 eq) was added under inert atmosphere. Subsequently, the reaction mixture was refluxed overnight. After cooling to ambient temperature, the potassium carbonate was filtered off and the solvent was evaporated under reduced pressure. The obtained residue was dissolved in dichloromethane (300 mL) and washed with distilled water (2 x 200 mL and brine (1 x 200 mL). The organic phase was dried over anhydrous $MgSO_4$, filtered and evaporated under reduced pressure to afford the crude product. A silica gel column chromatography (solid deposit) eluted with dichloromethane/methanol (9:1 → 8:2, $R_{f(DCM\ MeOH:8/2)}$ = 0,24) gave pure product **6** (34 %, 0.91 g, 2.01 mmol) as a yellow solid.

^{1}H NMR (500 MHz, $CDCl_3$): δ (ppm) 1.48-153 (m, 4H), 1.75-1.81(m, 2H), 1.93-199 (m, 2H), 3.91 (t, *J* = 6.3 Hz, 2H), 3.99 (t, *J* = 7.3Hz, 2H), 4.53 (d, *J* = 6.1 Hz, 2H), 5.10-5.13 (m, 1H), 5.21-5.26 (m, 1H), 5.89-5.95 (m, 1H), 6.96-7.03 (m, 2H), 7.13-7.18 (m, 2H), 7.31 (dd, *J* = 7.2 Hz and 2.4 Hz, 1H), 7.42 (t, *J* = 7.7 Hz, 2H), 7.93-8.01 (m, 2H), 8.30 (d, *J* = 8.6 Hz, 2H), 9.36 (bs, 1H), 10.32 (s, 1H).

^{13}C NMR (125 MHz, $CDCl_3$): δ (ppm) 25.7, 26.5, 29.0, 30.1, 49.0, 112.9, 118.7, 118.8, 119.0, 120.8, 123.0, 124.1, 124.9, 125.9, 128.9, 130.0, 133.1, 133.3, 141.3, 151.2, 152.3, 156.0, 190.4.

HRMS-FAB: theoretical $C_{29}H_{31}N_2O_3$ $[M+H]^+$ 455-2117, experimental 455.2115

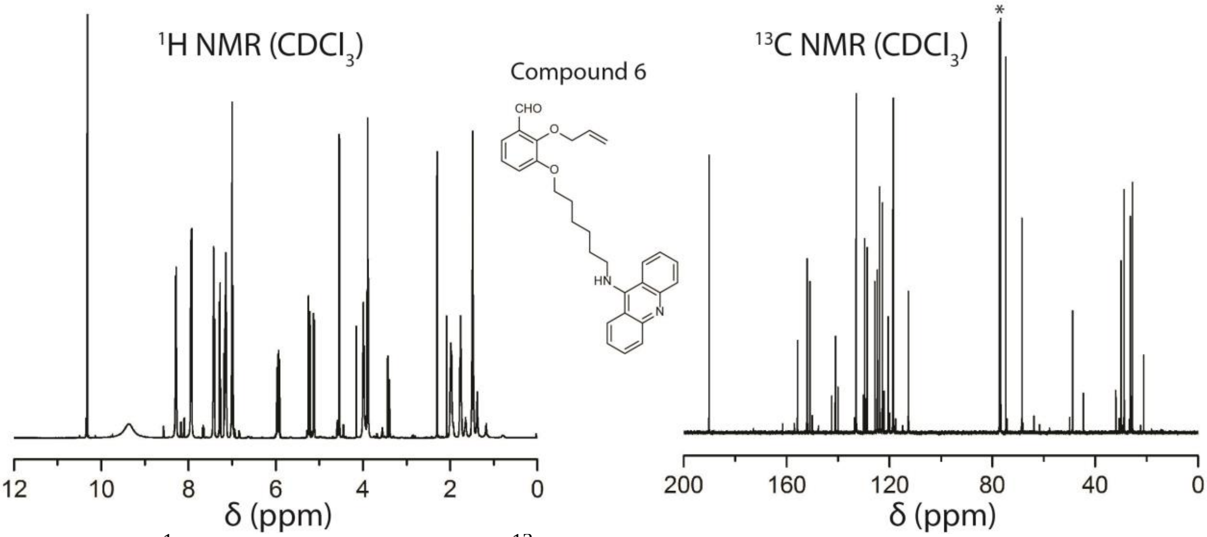

Figure S4: ^{1}H (500 MHz, $CDCl_3$) and ^{13}C (125 MHz, $CDCl_3$) NMR spectra of compound 6

3-((6-(acridin-9-ylamino)hexyl)oxy)-2-hydroxybenzaldehyde (compound 7)

A mixture of 3-((6-(acridin-9-ylamino)hexyl)oxy)-2-(allyloxy)benzaldehyde (**6**) (0.91 g,2.01 mmol, 1.0 eq), $Pd(OAC)_2$ (4.5 mg, 0.02 mmol, 0.01 eq) and PPh_3 (21 mg, 0.08 mmol, 0.04 eq) in 80% ethanol (10 mL) was stirred at ambient temperature for 10 minutes. Triethylamine (0.335 mL, 2.4 mmol, 1.2 eq) and formic acid (0.226 mL, 6.0 mmol, 3.0 eq) was added, and the reaction mixture was refluxed for additional 2 h. The solvent was evaporated under reduced pressure and the residue was dissolved in dichloromethane (40 mL). The organic phase was washed, respectively, with distilled water (2 x 25 mL) and brine (25 mL). The organic solvent was removed by rotary evaporator to afford a brown oil which was further purified by silica gel column chromatography (via solid deposition) using chloroform/methanol as eluent (98:2 → 95:5 → 9:1 → 8:2, $R_{f(CHCl3/MeOH:9/1)}$ = 0,25) to afford pure product **7** (62 %, 514 mg, 1.24 mmol) as an orange solid.

^{1}H NMR (500 MHz, $CDCl_3$): δ (ppm) 1.47-163 (m, 4H), 1.73-1.85 (m, 2H), 1.99-2.03 (m, 2H), 3.99 (t, *J* = 6.3 Hz, 2H), 4.07 (t, *J* = 7.3 Hz, 2H), 6.90 (t, *J* = 7.9 Hz, 1H), 7.05 (d, *J* = 7.3 Hz, 1H), 7.14-7.19 (m, 3H), 7.35-7.45 (m, 2H), 7.68-7.74 (m, 1H), 7.96-8.04 (m, 2H), 8.31-8.41 (m, 1H), 9.81 (bs, 1H), 9.89(s, 1H), 13.44 (bs, 1H).

^{13}C NMR (125 MHz, $CDCl_3$): δ (ppm) 25.1, 26.2, 28.7, 29.9, 48.5, 69.1, 119.2, 119.5, 20.9, 123.0, 124.5, 126.0, 128.0, 128.9, 130.5, 133.9, 135.5, 140.2, 147.5, 151.8, 156.8, 196.4

HRMS-FAB: theoretical $C_{26}H_{27}N_2O_3$ $[M+H]^+$ 415.2193, experimental 415.2191.

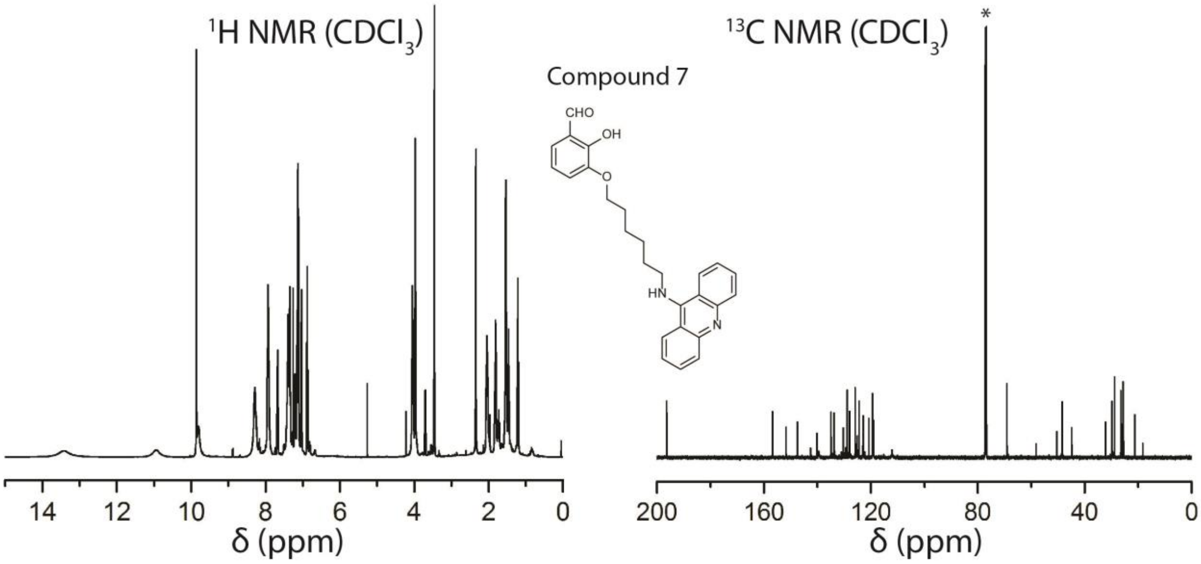

Figure S5: ^{1}H (500 MHz, $CDCl_3$) and ^{13}C (125 MHz, $CDCl_3$) NMR spectra of compound 7

Synthesis of UO_2-bis-acridine (BAU)

Uranyl-bis-acridine (**9**) was synthesized following la previously reported procedure [2]. IMPORTANT: Care should be taken while handing uranyl compound because of its toxicity and residual radioactivity. In a refluxing solution of 3-((6-(acridin-9-ylamino)hexyl)oxy)-2-hydroxybenzaldehyde (**7**) (439 mg, 1.06 mmol, 1.0 eq) in methanol (20 mL) was added a solution of 1,2-benzenediamine (**8**) (57.3 mg, 0.529 mmol, 0.5 eq) in methanol (5 mL) in a dropwise manner under inert atmosphere. After 1 h, uranyl acetate dihydrate ($UO_2(OAc)_2.2H_2O$) (225.13 mg, 0.529 mmol, 0.5 eq) was added, and the reaction mixture was refluxed for additional 20 min. After cooling to ambient temperature, the precipitate was filtered off and further washed with cold methanol to give UO_2-bis-acridine (**9**) (29 %, 179 mg, 0.153 mmol) as an orange solid.

^{1}H NMR (500 MHz, DMSO-*d6*): δ (ppm) 1.42-1.52 (m, 4H), 1.54-1.61 (m, 4H), 1.81-1.92 (m, 8H), 3.89-4.01 (m, 4H), 4.15-4.21 (m, 4H), 6.56-6.67 (m, 2H), 7.12 (d, *J* = 8.1 Hz, 1H), 7.18-7.21 (m, 1H), 7.33-7.43 (m, 6H), 7.47 (d, *J* = 8.1 Hz, 1H), 7.51-7.57 (m, 2H), 7.69-7.81 (m, 9H), 8.33-8.48 (m, 4H), 9.61 (s, 2H).

^{13}C NMR (125 MHz, DMSO-*d6*): δ (ppm) 25.8, 26.7, 28.4, 30.8, 55.3, 69.1, 116.4, 118.6, 120.7, 122.7, 124.8, 128.6, 129.1, 134.9, 138.0, 141.4, 147.2, 159.6, 156.1, 159.5, 159.8, 161.6, 167.0.

HRMS-FAB: theoretical $C_{58}H_{55}N_6O_6$ $[M+H]^+$ 1169.3916, experimental 1169.3914.

ESI-MS: theoretical: 1169.4685, experimental m/z 1169.4689.

2. Cross-linking of DNA origami via BAU tethering

Microscopic investigations of the complexes in lanes 1-4 of Figure 4 (main text) indicated DNA origami interaction due BAU-induced tethering (Figure S6). To understand the chemical composition of the observed aggregates, we performed energy dispersive X-ray spectroscopy (EDS). EDS analysis was carried out in STEM mode for higher spatial resolution, and using a double-tilt holder with a Beryllium specimen cradle for enabling higher signal sensitivity. For better light element detection, a recent windowless detector (X-MaxN 100TLE, Oxford instruments, UK) was employed. The presence of uranium and oxygen is indicative of DNA-bound BAU molecules, and the phosphor signal stems from the DNA backbone. The experimental parameters for EDS acquisitions were optimized for minimum electron beam damage to DNA.

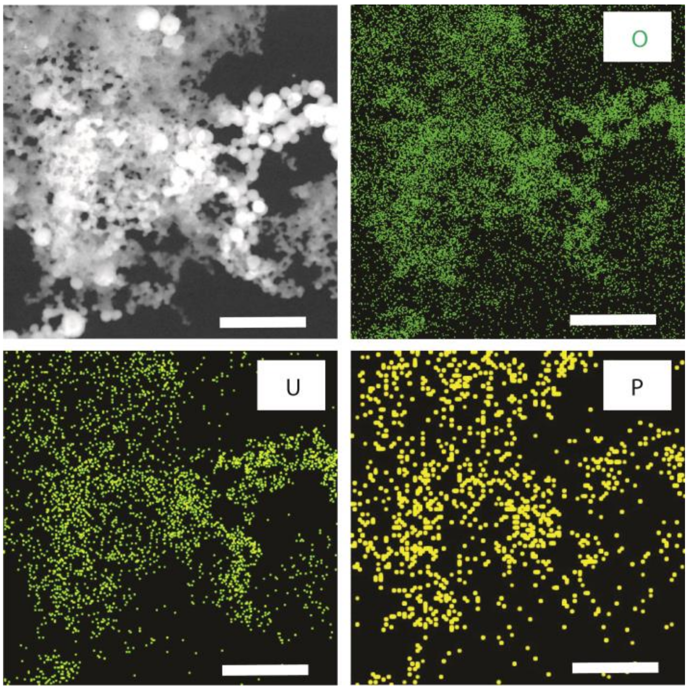

Figure S6: STEM imaging and EDS analysis indicates BAU-induced DNA origami cross-linking. Scale bar is 500 nm.

3. The effects of dye concentration and incubation time on the contrast of the stained DNA origami nanoplates

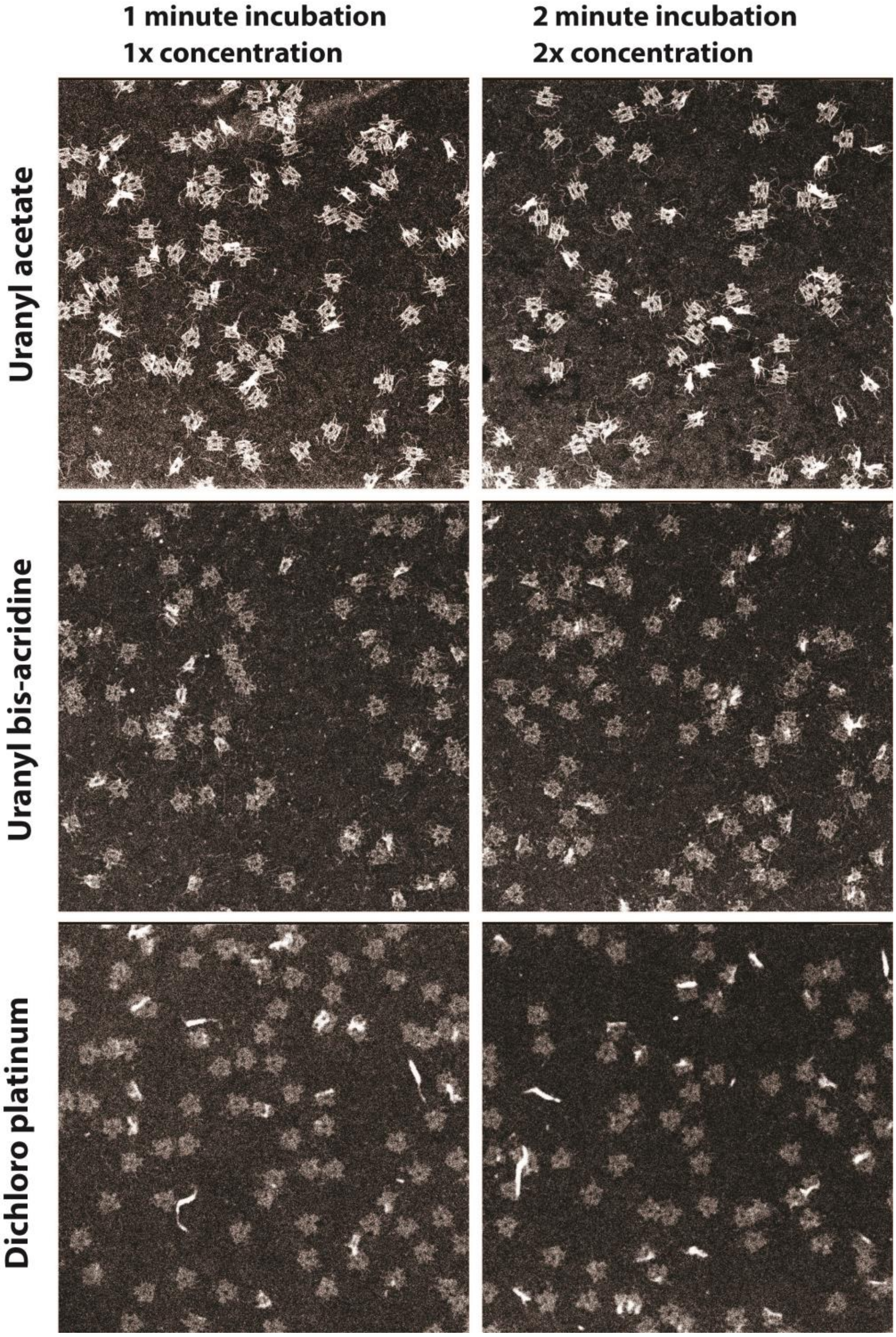

Figure S7: Contrast of the stained DNA origami saturates after 1 minute incubation time at 1x stock concentration of the electron dyes. Due to high binding affinity of the dyes, they react immediately with DNA (below 1 minute), after which the STEM contrast could not be increased anymore. The stock concentration (1x) for each dye is given in the experimental section.

4. Nanoparticle deposition onto TEM grids after incubation with cisplatin

After long incubation of 2 days with cisplatin, we observed very pronounced nanoparticle precipitation onto the TEM grids (panels a-b of Figure S8), where the clusters exhibited a dominant Cu/Pt signal in EDS elemental mapping (panels c-f, see also table S1 for quantitative composition analysis of the red dashed squares 1 and 2 in Figure S8b). Note that spurious X-rays caused by the stray electrons lead to uncertainty in distinguishing the Cu and Pt from one another. We could not detect any DNA origami nanoplates in the areas where Pt nanoparticles were absent, such as the marked region 2 in panel (b).

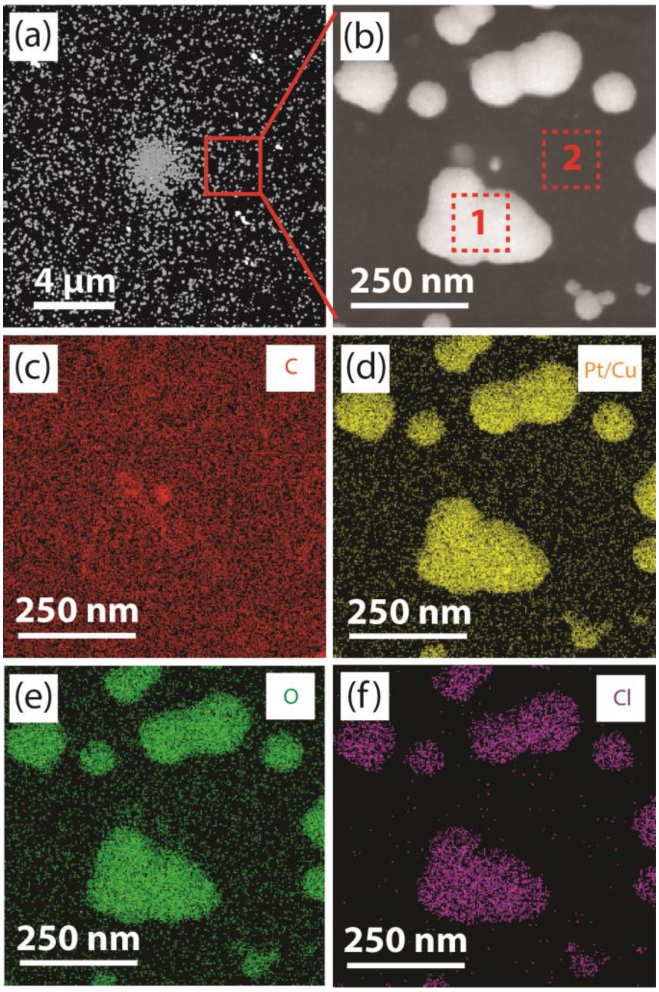

Figure S8: STEM imaging and EDS analysis demonstrate nanoparticle deposition onto TEM grids after in vitro staining of the DNA origami samples with cisplatin.

Table S1: EDS chemical analysis of marked regions 1 and 2 in Figure S8b.

Spectrum	C	Cu/Pt	O	Si	Cl	Other
1	23.1	40.8	25.9	1.1	6.2	2.9
2	81.3	7.6	6.5	4.6	-	-

References:

[1] C. J. Staveren, J. Eerden, F. C. J. M. Veggel, S. Harkema, D. N. Reinhoudt, *J Am Chem Soc* **1988**, *110*, 4994.
[2] D. M. Rudkevich, Z. Brzozka, M. Palys, H. C. Visser, W. Verboom, D. N. Reinhoudt, *Angew Chemie* **1994**, *106*, 480.
